# The positive association between hyperuricemia and polycystic ovary syndrome and analysis of related factors

**DOI:** 10.3389/fendo.2024.1356859

**Published:** 2024-06-12

**Authors:** Zhentao Gong, Yanwen Sun, Lingshan Zhang, Xiaoyong Zhu, Yingli Shi

**Affiliations:** ^1^ Department of Gynecology, Obstetrics and Gynecology Hospital of Fudan University, Shanghai, China; ^2^ Shanghai Medical College of Fudan University, Shanghai, China; ^3^ School of Global Public Health, New York University, New York, NY, United States; ^4^ Laboratory for Reproductive Immunology, Obstetrics and Gynecology Hospital of Fudan University, Shanghai, China; ^5^ Shanghai Key Laboratory of Female Reproductive Endocrine Related Diseases, Obstetrics and Gynecology Hospital of Fudan University, Shanghai, China

**Keywords:** polycystic ovary syndrome, hyperuricemia, hyperandrogenism, dyslipidemia, insulin resistance

## Abstract

**Purpose:**

To examine the potential association between polycystic ovary syndrome (PCOS) and hyperuricemia and to elucidate the underlying contributory factors.

**Methods:**

Retrospective study on 603 women with PCOS and 604 women without PCOS. Anthropometric features, reproductive hormone profiles, and metabolic parameters were measured and compared between two groups of patients. Examinations of correlations between SUA levels and other parameters were conducted to discern potential correlations.

**Results:**

Both serum uric acid levels and the incidence of hyperuricemia exhibited statistically significant elevations in women with PCOS when compared to their counterparts without PCOS. Nonetheless, this statistical difference was not found between the obese subgroup after stratifying study subjects by body mass index (BMI). Pearson’s correlation analysis underscored the prominence of BMI as a robust factor influencing SUA levels in women, regardless of their PCOS status. Furthermore, multivariable linear regression model demonstrated significant positive associations between SUA levels and several variables, namely dehydroepiandrosterone sulfate (DHEA-S), free androgen index (FAI), total cholesterol (TC), triglycerides (TG), free fatty acids (FFA), fasting insulin (FINS), homeostatic model assessment of insulin resistance (HOMA-IR), area under the curve for insulin (AUC-I), alanine aminotransferase (ALT), and aspartate aminotransferase (AST). Additionally, it is noteworthy that the prevalence of hyperuricemia exhibited a positive association with fasting plasma glucose (FPG) levels, while conversely, it displayed a negative association with estradiol (E2) levels.

**Conclusions:**

PCOS is associated with a significant elevation of SUA level and hyperuricemia prevalence. HA, IR, and dyslipidemia may be the mediators in the pathogenesis of hyperuricemia in women with PCOS.

## 1 Introduction

Polycystic ovary syndrome (PCOS) is the most common endocrine disorder in reproductive-aged women (1). According to various diagnostic criteria, PCOS is estimated to affect between 4–18% of women of reproductive age (2, 3). PCOS is a multifaceted disorder that encompasses a broad range of clinical manifestations beyond those related to the reproductive system. In particular, PCOS is frequently accompanied by metabolic disturbances, including abnormal lipid and glucose metabolism (4, 5), which contribute to an elevated risk of developing long-term complications such as cardiovascular diseases (6), diabetes (7), and cancer (8). Due to its high morbidity and the prevalence of associated complications, there is an urgent need for effective management and prevention of both short- and long-term sequelae.

Uric acid (UA) is a metabolic product derived from the oxidative degradation of purine compounds (9). Clinical diagnosis of hyperuricemia is typically established when serum uric acid (SUA) levels exceed 7.0 mg/dL (416 μmol/L) in men or 6.0 mg/dL (357 μmol/L) in women (10). Hyperuricemia is widely acknowledged not merely as a biomarker associated with various metabolic disorders, including obesity (11) and type 2 diabetes mellitus (T2DM) (12), but also as a predictor of hypertension, cardiovascular morbidity (13).

During our clinical practice, we observed that women with PCOS often present with elevated SUA levels. This observation raised our curiosity as to whether there is a correlation between elevated SUA levels/prevalence of hyperuricemia and PCOS. And if so, what are the contributing factors to this association? To answer this question, we conducted this retrospective study.

The objectives of this investigation encompass the examination of a plausible association between hyperuricemia and PCOS, with a concurrent effort to elucidate the underlying contributory factors. In undertaking this research, our overarching aim is to augment the existing comprehension of the complications associated with PCOS, thereby underscoring the importance of exploring a more comprehensive strategy for the effective management of this syndrome.

## 2 Methods

### 2.1 Study subjects

This retrospective cross-sectional study recruited 603 women with PCOS from the Reproductive Endocrinology Clinic, 604 women without PCOS but underwent surgical treatment for benign ovarian cysts from the Department of Gynecology, 1207 in total from Obstetrics and Gynecology Hospital of Fudan University between May 2021 and March 2023.

PCOS was diagnosed according to the Rotterdam criteria (14), when at least two of the following three criteria are met: 1) Oligo- or anovulation, 2) Presence of clinical and/or biochemical signs of hyperandrogenism (HA), and 3) Evidence of polycystic ovaries after exclusion of other potential etiologies such as congenital adrenal hyperplasia, androgen-secreting tumors, and Cushing’s syndrome. None of the participants in this study had administrated medications known to influence reproductive and metabolic processes during the six months prior to their enrollment.

The ethics committee of Obstetrics and Gynecology Hospital of Fudan University reviewed and approved this study protocol (kyy2023–68). The study protocol conforms rigorously to the ethical guidelines of the 1975 Declaration and its later amendments.

### 2.2 Assessments and definitions

This retrospective study involved the systematic collection and analysis of data pertaining to several key variables. These variables included body mass index (BMI), menstrual cycle characteristics, including duration and timing of the last three menstrual periods, as well as observable symptoms such as hirsutism, acne, alopecia, etc. Data, such as the levels of reproductive hormones, were collected through a thorough review of medical records from a selected population of patients who had previously sought medical attention for these conditions.

The body weight and height of participants were measured using standard protocol by skilled and experienced nurses. Subsequently, BMI was derived by dividing the measured body weight in kilograms by the square of the measured body height in meters. According to the computed value, BMI categories consisted of underweight (BMI<18.5 kg/m^2^), normal weight (18.5≤BMI<25kg/m^2^), overweight (25≤BMI<30kg/m^2^), and obese (BMI≥30kg/m^2^).

All participants underwent blood testing in the morning for reproductive hormones, sex hormone binding globulin (SHBG), lipids, alanine aminotransferase (ALT), aspartate aminotransferase (AST), UA and creatinine (Cr) during the follicular phase. The study also employed the oral glucose tolerance test (OGTT) and insulin release test (IRT) on participants who underwent an overnight fast of at least 8 hours prior to the test. Glucose and insulin levels were measured at various time intervals, including the basal sample, as well as at 0.5, 1, 2, and 3-hour intervals following the administration of glucose. Following sample collection, the specimens were stored at a temperature of 4°C and promptly conveyed to the clinical laboratory.

Hyperuricemia is characterized by a SUA level of at least 6mg/dL (357μmol/L) in women (10). The assessment of estimated glomerular filtration rate (eGFR) was based on the Chronic Kidney Disease-Epidemiology Collaboration formula (15) for White/other (except Black) expressed in milliliters per minute per 1.73 m^2^: Cr≤ 0.7 mg/dL, eGFR = 144 * (Cr/0.7)^-0.329^ *(0.993)^age^; Cr> 0.7 mg/dL, eGFR = 144 * (Cr/0.7)^-1.209^ *(0.993)^age^. Insulin resistance was assessed utilizing the homeostatic model assessment of insulin resistance (HOMA-IR). HOMA-IR = fast plasma glucose (FPG, mmol/L) * fasting serum insulin (FINS, mIU/mL)/22.5. The area under the insulin release curve (AUC-I) was calculated with the use of the trapezoidal method based on the results of the insulin-releasing test. Free androgen index (FAI%) = testosterone (T, ng/mL) ×100/SHBG (nmol/L).

### 2.3 Statistical analysis

Continuous variables were reported as medians with interquartile ranges, and categorical variables were presented as proportions (%). To account for skewed distribution, logarithmic transformation was applied to certain variables prior to statistical analysis. The statistical comparison of continuous variables was conducted using Student’s t-tests, while χ2 tests were used to compare categorical variables. Pearson correlation coefficient was employed to examine the potential associations between SUA levels and baseline characteristics index such as age, BMI and eGFR. Furthermore, multivariable linear regression model was utilized to assess the relationships between the variables while controlling pertinent confounding variables.

All statistical analyses were conducted using R version 4.2.1 (R Foundation for Statistical Computing, Vienna, Austria, www.R-project.org). A two-tailed test was utilized to ascertain statistical significance, and a P-value less than 0.05 was regarded as statistically significant. Statistical graphs are plotted using GraphPad Prism version 9.0.0 for Windows (GraphPad Software, Boston, Massachusetts USA, www.graphpad.com).

## 3 Results

### 3.1 Baseline characteristics

The baseline characteristics of PCOS and non-PCOS subjects were compared and presented in **Table 1**. The results showed that women in PCOS group were younger (P < 0.001) and had higher BMI (P < 0.001). The median SUA level was 345.25 μmol/L, and the prevalence of hyperuricemia was 38.97% in the PCOS group, which were significantly higher than those in the non-PCOS group with median SUA level of 280.49 μmol/L (*P* < 0.001) and prevalence of hyperuricemia of 9.77% (*P* < 0.001). We also found that the levels of hormones including T (*P* < 0.001), dehydroepiandrosterone sulfate (DHEA-S) (*P* < 0.001), luteinizing hormone (LH) (*P* < 0.001), LH/FSH (*P* < 0.001) and anti-Müllerian hormone (AMH) (*P* < 0.001) were significantly higher while estradiol (E2) (*P* < 0.001) and progesterone (P) (*P* < 0.001) were lower in PCOS group. No significant difference in the follicle stimulating hormone (FSH) (*P* > 0.05) and prolactin (PRL) (*P* > 0.05) levels were observed between the two groups.

**Table 1:** Baseline characteristics of the study subjects.

Variable	PCOS		NON-PCOS		*P* value
	n	x ± SD	n	x ± SD	
Age (years)	603	25.64 ± 4.74	604	28.07 ± 4.40	< 0.001
BMI (kg/m(2))	603	25.04 ± 5.01	604	22.00 ± 3.57	< 0.001
E2 (ng/mL)	367	63.88 ± 57.76	598	101.40 ± 85.84	< 0.001
P (ng/mL)	262	1.17 ± 2.44	502	2.32 ± 4.23	< 0.001
T (ng/mL)	603	0.77 ± 0.27	532	0.52 ± 0.21	< 0.001
DHEA-S (μg/dL)	561	303.20 ± 126.01	435	265.40 ± 107.20	< 0.001
FSH (mIU/mL)	434	6.73 ± 1.91	599	6.64 ± 3.20	> 0.05
LH (mIU/mL)	568	12.30 ± 7.54	596	9.86 ± 12.76	< 0.001
PRL (ng/mL)	410	13.61 ± 8.20	515	14.65 ± 10.10	> 0.05
AMH (ng/mL)	369	8.26 ± 4.41	569	4.04 ± 2.45	< 0.001
LH/FSH	433	1.83 ± 1.02	596	1.40 ± 1.11	< 0.001
FPG (mmol/L)	594	4.96 ± 0.81	588	4.94 ± 0.47	> 0.05
TC (mmol/L)	555	4.84 ± 0.98	589	4.61 ± 0.81	< 0.001
TG (mmol/L)	555	1.53 ± 1.31	589	0.94 ± 0.56	< 0.001
HDL (mmol/L)	544	1.31 ± 0.66	587	1.47 ± 0.34	< 0.001
LDL (mmol/L)	554	3.18 ± 0.83	587	2.91 ± 0.72	< 0.001
Apo-A (g/L)	418	1.29 ± 0.28	564	1.43 ± 0.24	< 0.001
Apo-B (g/L)	418	0.94 ± 0.32	564	0.80 ± 0.22	< 0.001
Lpa (mg/L)	413	203.31 ± 240.47	563	174.06 ± 214.51	< 0.05
FFA (mmol/L)	416	0.61 ± 0.28	563	0.55 ± 0.25	< 0.001
eGFR (mL/min/1.73m(2))	602	145.89 ± 26.14	604	149.19 ± 25.79	< 0.05
ALT (U/L)	598	28.09 ± 32.44	503	15.16 ± 10.47	< 0.001
AST (U/L)	361	24.76 ± 21.22	603	17.55 ± 5.50	< 0.001
Uric acid (μmol/L)	603	345.25 ± 81.96	604	280.49 ± 64.14	< 0.001
Hyperuricemia (%)	603	38.97	604	9.77	< 0.001

E2, estradiol; P, progesterone; DHEA-S, dehydroepiandrosterone sulfate; FSH, follicle-stimulating hormone; LH, luteinizing hormone; PRL, prolactin; AMH, anti-Müllerian hormone; TC, total cholesterol; TG, triglyceride; HDL, high-density lipoprotein; LDL, low-density lipoprotein; Apo-A, Apolipoprotein A; Apo-B, Apolipoprotein B; Lpa, Lipoprotein(a); FFA, free fatty acid.

**Figure 1:**
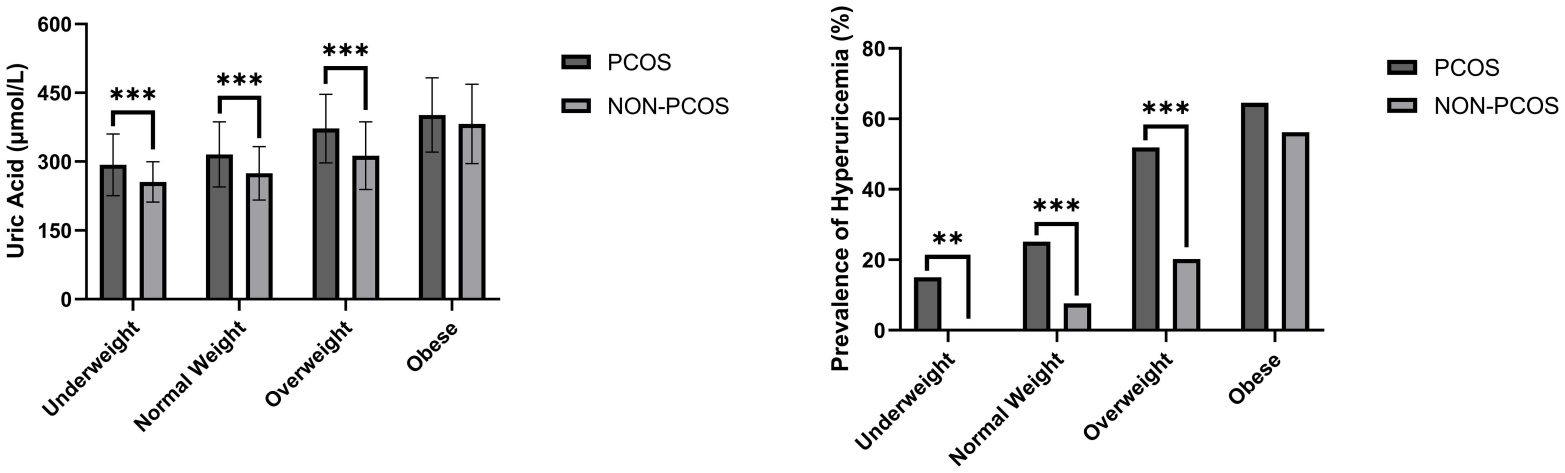
SUA level and prevalence of hyperuricemia comparisons between women with/without PCOS stratified based on BMI. **P < 0.01; ***P < 0.001.

In addition, women in PCOS group had higher levels of total cholesterol (TC) (*P* < 0.001), triglyceride (TG) (*P* < 0.001), free fatty acids (FFA) (*P* < 0.001), low-density lipoprotein (LDL) (*P* < 0.001), apolipoprotein-B (Apo-B) (*P* < 0.001), Lpa (*P* < 0.05), ALT (*P* < 0.001), AST (*P* < 0.001), and lower apolipoprotein-A (Apo-A) (*P* < 0.001), high-density lipoprotein (HDL) (*P* < 0.001) and eGFR (*P* < 0.01) compared to non-PCOS group. No significant difference in the level of FPG was observed. After stratifying the study subjects based on BMI categories, the SUA levels and the prevalence of hyperuricemia were compared and presented in **Figure 1**, and the baseline characteristics of all four subgroups of PCOS were also compared and presented in **Table 2**.

**Table 2:** Baseline characteristics of all four subgroups of PCOS subjects stratified based on BMI.

Variable	Underweight		Normal Weight		Overweight		Obese		*P* value
	n	x ± SD	n	x ± SD	n	x ± SD	n	x ± SD	
Age (years)	40	23.53 ± 4.46	282	25.07 ± 4.65	185	26.56 ± 4.60	96	26.44 ± 4.96	a*; b**
BMI (kg/m^2^	40	17.58 ± 0.98	282	21.77 ± 1.71	185	27.20 ± 1.40	96	33.58 ± 2.93	a, b, c***
E2 (ng/mL)	27	93.33 ± 103.37	160	70.30 ± 68.56	115	53.18 ± 29.25	65	54.77 ± 27.84	b**
P (ng/mL)	18	1.31 ± 2.66	111	1.30 ± 2.81	80	1.14 ± 2.02	53	0.89 ± 2.12	> 0.05
T (ng/mL)	40	0.76 ± 0.25	282	0.78 ± 0.28	185	0.75 ± 0.26	96	0.82 ± 0.24	c*
DHEA-S (μg/dL)	40	296.66 ± 135.57	261	300.52 ± 117.81	170	302.35 ± 137.94	90	315.47 ± 122.42	> 0.05
FSH (mIU/mL)	30	7.93 ± 3.30	189	6.98 ± 1.88	141	6.27 ± 1.63	74	6.51 ± 1.42	b***
LH (mIU/mL)	38	16.68 ± 8.23	263	13.66 ± 8.74	176	10.43 ± 5.58	91	10.19 ± 4.68	a*; b***; c*
PRL (ng/mL)	29	14.68 ± 7.14	187	14.31 ± 8.30	128	13.03 ± 9.29	66	12.28 ± 5.55	> 0.05
AMH (ng/mL)	23	8.39 ± 3.63	154	9.66 ± 5.08	128	7.47 ± 3.54	64	6.46 ± 3.41	b***
SHBG (nmol/L)	29	67.57 ± 29.04	169	51.88 ± 38.95	121	27.11 ± 23.46	67	23.67 ± 16.07	a*; b***
FAI (%)	29	1.28 ± 0.70	169	2.20 ± 1.50	121	4.73 ± 8.81	67	5.56 ± 7.44	a***; b**
LH/FSH	30	2.27 ± 1.07	189	2.05 ± 1.18	140	1.59 ± 0.78	74	1.54 ± 0.73	b**
TC (mmol/L)	36	4.52 ± 0.65	259	4.80 ± 0.98	168	4.89 ± 1.00	92	5.02 ± 1.04	> 0.05
TG (mmol/L)	36	0.95 ± 0.86	259	1.22 ± 0.86	168	1.95 ± 1.86	92	1.88 ± 0.93	b***
HDL (mmol/L)	36	1.49 ± 0.39	251	1.43 ± 0.92	166	1.17 ± 0.21	91	1.17 ± 0.24	b***
LDL (mmol/L)	36	2.72 ± 0.56	259	3.08 ± 0.82	167	3.27 ± 0.80	92	3.50 ± 0.86	a, c*
Apo-A (g/L)	22	1.43 ± 0.34	196	1.35 ± 0.30	124	1.22 ± 0.22	76	1.21 ± 0.25	b***
Apo-B (g/L)	22	0.75 ± 0.10	196	0.86 ± 0.22	124	1.00 ± 0.38	76	1.07 ± 0.38	a, b***
Lpa (mg/L)	22	175.95 ± 289.35	195	247.19 ± 281.43	124	171.44 ± 185.25	72	147.73 ± 156.47	b**
FFA (mmol/L)	22	0.73 ± 0.24	197	0.59 ± 0.25	124	0.59 ± 0.19	73	0.65 ± 0.45	a*
eGFR (mL/min/1.73m^2^)	40	145.43 ± 23.74	282	144.80 ± 26.62	184	148.60 ± 26.03	96	144.10 ± 25.89	> 0.05
FPG (mmol/L)	39	4.61 ± 0.41	280	4.77 ± 0.42	181	5.19 ± 1.00	94	5.23 ± 1.17	a*; b***
FINS (μIU/mL)	39	6.44 ± 2.72	276	8.56 ± 4.30	178	16.10 ± 8.84	92	21.52 ± 14.35	a, b, c***
HOMA-IR	39	1.34 ± 0.62	276	1.85 ± 1.02	178	3.89 ± 2.87	92	5.22 ± 4.86	a, b***; c**
AUC-I (μIU*h/mL)	34	135.54 ± 64.32	242	195.55 ± 121.75	157	289.64 ± 157.95	73	325.34 ± 139.92	a, b***
ALT (U/L)	40	15.50 ± 10.25	278	17.96 ± 13.22	185	33.88 ± 36.97	95	51.73 ± 49.47	a, b***; c**
AST (U/L)	28	23.39 ± 29.08	160	19.49 ± 6.74	117	26.92 ± 26.11	56	35.98 ± 27.23	b**; c*
Uric acid (μmol/L)	40	293.08 ± 67.36	282	315.81 ± 71.13	185	372.17 ± 74.79	96	401.59 ± 81.04	b***; c**
Hyperuricemia (%)	40	15.00	282	25.18	185	51.89	96	64.58	b***; c*

The data are presented as the mean ± standard deviation.

a Significant difference between underweight group and normal weight group.

b Significant difference between normal weight group and overweight group.

c Significant difference between overweight group and obese group.

*P < 0.05; **P < 0.01; ***P < 0.001.

*, P < 0.05; **, P < 0.01; ***, P < 0.001

### 3.2 Associations between age, BMI, eGFR and SUA levels in PCOS populations

We discovered significant differences in the mean levels of SUA and the prevalence of hyperuricemia among underweight (*P* < 0.001), normal weight (*P* < 0.001) and overweight subgroups (*P* < 0.001) after stratifying the study population based on BMI. In other words, there were no statistically significant differences between SUA levels and the occurrence of hyperuricemia in the obese subgroup (**Table 3**).Additionally, the levels of SUA were found to be correlated with BMI in Pearson’s correlation analysis (*r* = 0.460, *p* < 0.001), however, its correlations with age and eGFR were not statistically significant.

**Table 3:** Correlations between the levels of age, BMI, eGFR and serum uric acid.

Baseline Characteristics	Coefficient	
	*r*	*P*
Age	0.039507	0.332797
BMI	0.460060^***^	6.4588E-33
eGFR	-0.078532	0.054130

***P < 0.001.

### 3.3 Associations between the levels of reproductive hormones and SUA in PCOS populations

Multivariable linear regression model revealed that both SUA level and the prevalence of hyperuricemia were positively associated with the levels of DHEA-S (b = 0.082, P < 0.001; b = 0.002, P < 0.05) and FAI (b = 9.209, P < 0.001; b = 0.245, P < 0.001). And the prevalence of hyperuricemia was negatively associated with the E2 level (b = -0.007, P < 0.05).

**Figure 2:**
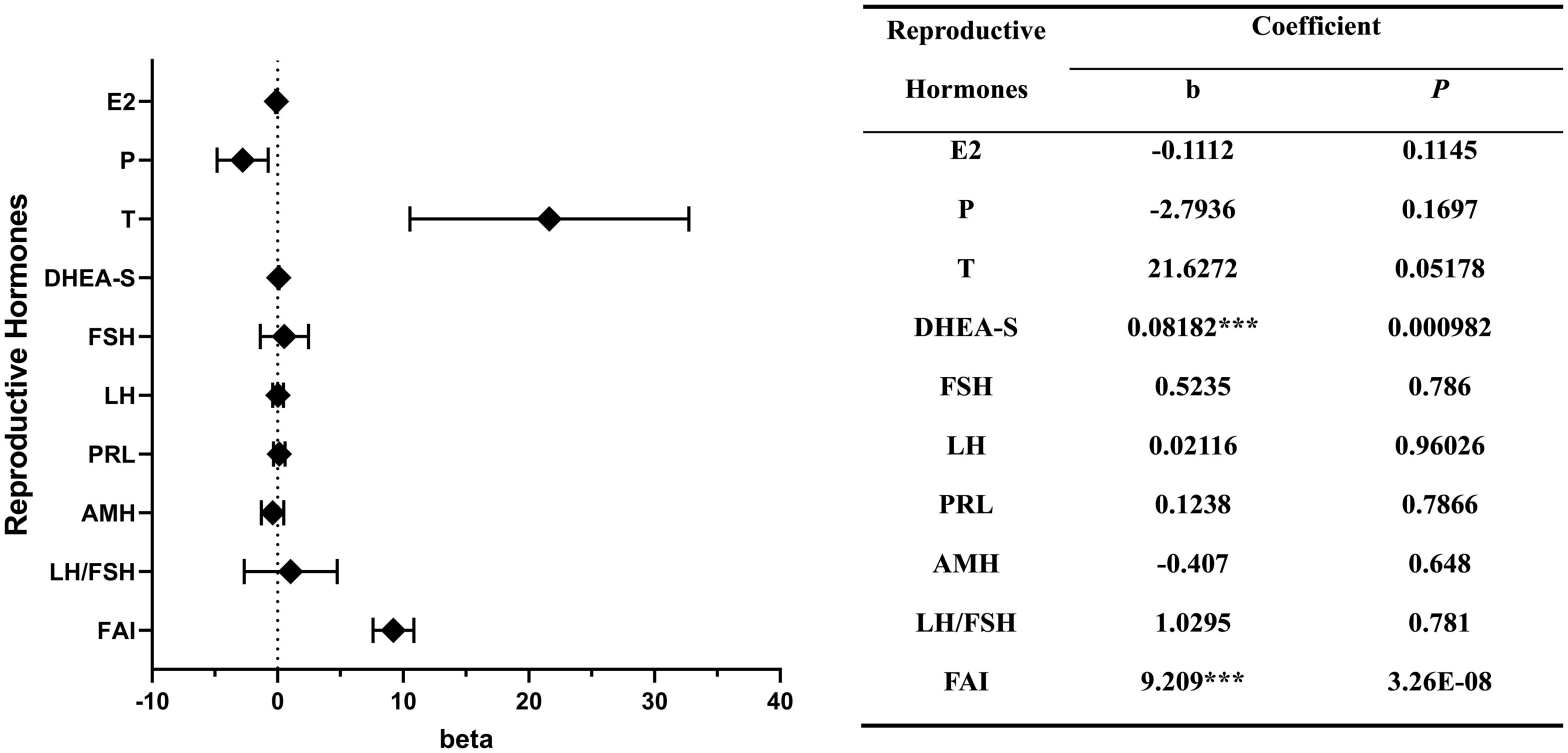
Correlations between the levels of reproductive hormones and serum uric acid. ***P < 0.001.

SUA levels were also observed to have a positive association with the levels of LH, FSH, LH/FSH, PRL and T, and a negative association with the levels of E2, P and AMH. However, these associations did not reach statistical significance (**Figure 2**).

*, P < 0.05; **, P < 0.01; ***, P < 0.001

### 3.4 Associations between the levels of metabolic indexes and SUA in PCOS populations

Multivariable linear regression model also demonstrated a significant positive association between SUA levels and levels of TC (b = 6.378, P < 0.05), TG (b = 11.467, P < 0.001), LDL (b = 8.250, P < 0.05), FFA (b = 43.922, P < 0.001), Apo-A (b = 28.388, P < 0.05), Apo-B (b = 30.851, P < 0.01), FINS (b = 1.206, P < 0.01), HOMA-IR (b = 2.633, P < 0.05), AUC-I (b = 0.099, P < 0.001), ALT (b = 0.532, P < 0.001) and AST (b = 1.045, P < 0.001).

In the context of examining the relationship between the prevalence of hyperuricemia and various metabolic parameters, we observed that TC exhibited a statistically significant positive correlation (b = 0.194, P < 0.05), as did TG (b = 0.356, P < 0.001), FFA (b = 1.373, P < 0.01), FINS (b = 0.042, P < 0.01), HOMA-IR (b = 0.102, P < 0.05), AUC-I (b = 0.003, P < 0.001), ALT (b = 0.015, P < 0.001) and AST (b = 0.051, P < 0.001) also displayed a positive correlation.

**Figure 3:**
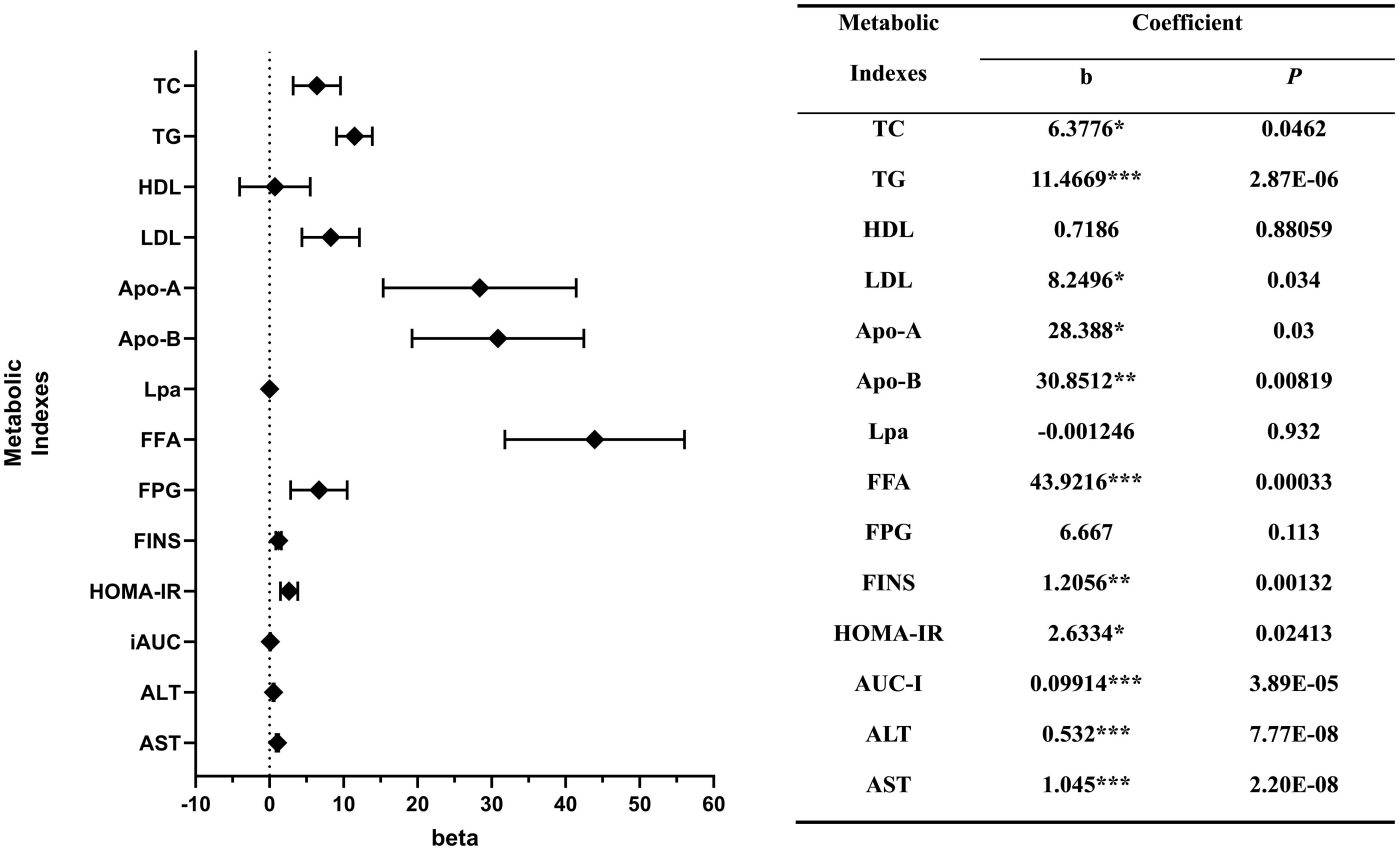
Correlations between the levels of metabolic indexes and serum uric acid. *P < 0.05; **P < 0.01; ***P < 0.001.

The associations between the levels of FPG (b = 0.297, P < 0.05) and the prevalence of hyperuricemia is statistically significant, while their association with the level of SUA did not reach statistical significance. SUA level also had a positive association with the level of HDL and a negative association with the level of Lpa. However, these associations did not reach statistical significance (**Figure 3**).

*, P < 0.05; **, P < 0.01; ***, P < 0.001

## 4 Discussion

To the best of our knowledge, this is the first study in women with PCOS demonstrating a significant association between HA, IR, dyslipidemia, and hyperuricemia. Through our investigation into the interplay of these pathologies, we have unveiled the association of PCOS, hyperuricemia and its long-term complexities. These findings underscore the imperative for a more comprehensive therapeutic strategy in addressing the multifaceted challenges posed by this condition. The discerned associations and their underlying mechanisms not only enhance our theoretical grasp of PCOS but also provide promising avenues for the potential development of supplementary therapeutic interventions.

Inspired by observation from clinical practice that women with PCOS tended to be combined with higher level of SUA and cognizant of the consequential health risks entailed by hyperuricemia, we conducted this retrospective study. The primary objective of this study was to examine the potential existence of a correlation between hyperuricemia and PCOS, with a concomitant focus on delineating the specific contributing factors that underpin this relationship.

This study revealed that there exist statistically significant differences between the SUA level and the prevalence of hyperuricemia between PCOS group and non-PCOS group. However, after stratifying the study population based on BMI, this difference become insignificant among obese group. Pearson’s correlation analysis also suggests a positive correlation between the level of BMI and SUA. This result further stresses the strong correlation between high BMI and elevated level of SUA. Considering it, we include it in the multiple linear regression model as a confounder to the SUA level. Although no statistical significance was found between age, eGFR and SUA levels in this research, numerous retrospective studies (16–19) had established solid correlations between age, eGFR and SUA levels. As such, we conducted the subsequent analysis with stratification based on these two factors along with BMI.

Correlation analyses based on multivariable linear regression model showed that, in PCOS group, both SUA level and prevalence of hyperuricemia are positively correlated to the level of DHEA-S, FAI, TC, TG, FFA, FINS, HOMA-IR, AUC-I, ALT and AST. Meanwhile, SUA level is also positively correlated to the levels of LDL, Apo-A and Apo-B; the prevalence of hyperuricemia is negatively correlated to the level of E2 and positively correlated to the level of FPG. These discoveries indicate that higher levels of SUA and prevalence of hyperuricemia are highly associated with HA, IR, dyslipidemia and potential hypohepatia in women with PCOS, and E2 may serve as a protective factor.

A plasma metabolomics analysis demonstrated that among several sub-classifications of PCOS, the HA subtype exhibited significantly elevated levels of SUA when compared to the oligo-/anovulation and PCOM subtypes (20), which indicates that there might exist a positive correlation between the levels of androgen and SUA in women with PCOS. Numerous studies have indicated positive correlations between either T or DHEA-S and SUA, though not completely identical (21–25). The outcomes of our study support this proposed correlation as we have identified a statistically significant association between DHEA-S, FAI levels and SUA levels. It is noteworthy that the correlation between T and SUA levels, while not achieving statistical significance (*p* = 0.052), exhibits a notable degree of proximity to significance in our findings. The presence of a robust correlation between the levels of androgens and UA remains ambiguous, however, existed researches suggest that androgens could elevate SUA levels through the stimulation of hepatic purine nucleotide metabolism (26) and enhance the reabsorption of UA in the kidney (27). In addition to this, we also observed a negative association between the levels of E2 and SUA, we suggest that it may be attributed to the capacity of E2 to lower SUA levels by stimulating UA excretion in the renal system (28).

Mu et al. undertook a retrospective inquiry aiming to explore the relationship between hyperuricemia prevalence and reproductive hormones in PCOS (23). The findings of the study suggested a strong association between T levels and the levels of SUA. Beyond the purview of sex hormones, PCOS encompasses series of metabolic disorders such as IR and dyslipidemia. However, these were not considered in the study by Mu et al. In addition to this, as an important aspect of PCOS, although HA exerts a substantial influence across various pathogenic mechanisms inherent to the syndrome, whether it is significantly related to hyperuricemia is conflict (22, 24, 27, 29). This controversy also raised our interest, prompting a systematic exploration into the potential presence of confounding variables that might influence SUA levels among individuals with PCOS. Consequently, our research endeavors to address this void by examining not only the relationship between SUA and reproductive hormones, but also the role of lipids, insulin, etc. By doing so, we aim to provide a more comprehensive and convincing analysis of the association between hyperuricemia and PCOS.

It has been extensively reported that SUA levels are strongly associated with the degree of IR (30–32). Given that IR is a hallmark feature of PCOS, it was anticipated that there would exist a significant positive correlation between SUA levels and either HOMA-IR or AUC-I in PCOS patients. Our study findings supported this hypothesis, as we identified significant positive associations between SUA levels and FPG, FINS, HOMA-IR, and AUC-I. One plausible mechanism underlying this relationship could be the insulin’s potential role in promoting renal urate reabsorption by activating URAT1 and the sodium-dependent anion co-transporter located in the brush border membranes of the renal proximal tubule (33).

Moreover, a prospective study conducted on a large population also discovered a bell-shaped curve relationship between SUA and glucose levels (34). Specifically, when glucose levels surpass a certain threshold, which was determined to be eight mmol/L in that study, SUA levels decline as glucose levels increase. Conversely, when glucose levels are below this threshold, SUA levels tend to increase as glucose levels increase. However, we did not observe this bell-shaped relationship in our study. A potential explanation for this disparity could be the participants included in this investigation were of a relatively youthful age and tended to display less pronounced severity in their glucose metabolism disorders.

In addition to insulin, lipids also play a significant role in the pathogenesis of hyperuricemia. Rats that were fed a high-fat diet and subsequently developed obesity exhibited increased activity of XOR in their subcutaneous adipose tissue (35). Furthermore, the accumulation of UA in the body triggers *de novo* lipogenesis, thereby facilitating the synthesis of fatty acids within the liver (36). This process establishes a positive feedback loop between dyslipidemia and hyperuricemia. Our findings suggest that the aforementioned self-sustaining vicious cycle predominantly contributes to the metabolic derangement of lipids, particularly free fatty acids, which emerges as the most notably associated factor. This finding also provides valuable insight, indicating that interventions aimed at reducing lipid levels could be greatly beneficial for PCOS patients with coexisting hyperuricemia.However, this study inevitably has its limits. The research design is fundamentally retrospective, thereby limiting our ability to gather blood samples and data at varying intervals over an extended period. To maintain stability in the plasma levels of reproductive hormones, we collected blood samples exclusively during the follicular phase, when basal levels of reproductive hormones were assessed, without dynamic monitoring. Due to these constraints, we were unable to conduct a cohort study similar to the one conducted by Mumford et al. (37) This limitation might have constrained the demonstration of the correlation between E2 and UA levels. Our comparison of baseline characteristics between the PCOS and control groups revealed that E2 and P levels were significantly lower in the PCOS group compared to the control group, while this trend was opposite to the differences observed in SUA levels between the two groups. However, in the correlation analysis conducted using a multivariable linear regression model, no significant association was found between E2 or P levels and SUA levels. In fact, one study suggests that uric acid levels in follicular fluid may influence follicle development by promoting oxidative stress (38). The discrepancies between our study and the aforementioned research warrant further investigation through subsequent cohort studies and additional animal experiments. Moreover, in this study, we included women aged 18 to 35 years, which is a relatively narrow age range. Therefore, it is important to recognize that the correlations we have identified might have limitations due to the restricted age group we focused on.

Conducting subgroup analyses comparing PCOS and non-PCOS populations would undoubtedly enhance the statistical robustness of our study. However, upon stratification, we observed a significantly uneven distribution among the four groups, particularly within the obese subgroup (96/19), indicating pronounced disparities. Furthermore, we detected instances of heteroscedasticity. These issues, compounded by substantial discrepancies in sample sizes, led us to abstain from incorporating these results into the study. Nevertheless, this decision may render the results contentious and potentially preclude the derivation of more nuanced conclusions.

To obtain a compelling conclusion from a cross-sectional study, it is imperative to have a sufficiently large sample size. Unfortunately, the present study was unable to collect a dataset of adequate magnitude. For example, the reported bell-shaped curve relationship between FPG and SUA levels was not found in this study because of the limited sample size. Furthermore, conducting studies in a single center may lead to selection bias, which can further compromise the validity and generalizability of the results. The imperative for further research necessitates a multicenter study, characterized by a larger sample size to enhance the robustness and generalizability of our findings.

## 5 Conclusions

To conclude, this study demonstrated that PCOS is associated with a significant elevation of SUA level and hyperuricemia prevalence. A higher level of DHEA-S, FAI, TC, TG, FFA, FINS, HOMA-IR, AUC-I, ALT, and AST were strongly correlated with both elevated SUA level and hyperuricemia prevalence, suggesting that HA, IR, and dyslipidemia may be the mediators in the pathogenesis of hyperuricemia in women with PCOS. Through the elucidation of these associations, we gained a deeper understanding of the complexity of PCOS and its long-term effects on multiple organ systems, which provides us with an important and sufficient foundation for its early diagnosis and early management.

## Data availability statement

The raw data supporting the conclusions of this article will be made available by the authors, without undue reservation.

## Ethics statement

The studies involving humans were approved by the Ethics Committee of the Obstetrics and Gynecology Hospital of Fudan University. The studies were conducted in accordance with the local legislation and institutional requirements. Written informed consent for participation was not required from the participants or the participants’ legal guardians/next of kin in accordance with the national legislation and institutional requirements.

## Author contributions

ZG: Investigation, Methodology, Visualization, Writing – original draft, Writing – review & editing. YWS: Methodology, Visualization, Writing – review & editing. LZ: Writing – review & editing. XZ: Funding acquisition, Supervision, Writing – review & editing. YLS: Conceptualization, Funding acquisition, Supervision, Writing – review & editing.
